# Discourses of student orientation to medical education programs

**DOI:** 10.3402/meo.v19.23714

**Published:** 2014-03-14

**Authors:** Rachel H. Ellaway, Gerry Cooper, Tracy Al-Idrissi, Tim Dubé, Lisa Graves

**Affiliations:** 1 Undergraduate Medical Education, Northern Ontario School of Medicine, Greater Sudbury, ON, Canada; 2 Schulich School of Medicine & Dentistry – Windsor Program, Western University, Windsor, ON, Canada; 3 Office of the Registrar, Trent University, Peterborough, ON, Canada; 4 Department of Family and Community Medicine, St. Michael's Hospital, Toronto, ON, Canada

**Keywords:** medical student orientation, white coat ceremony, professionalism, medical culture, transition, identity development

## Abstract

**Background:**

Although medical students’ initial orientation is an important point of transition in medical education, there is a paucity of literature on the subject and major variations in the ways that different institutions orient incoming medical students to their programs.

**Methods:**

We conducted a discourse analysis of medical education orientation in the literature and on data from a survey of peer institutions’ approaches to orientation.

**Results:**

These two discourses of orientation had clear similarities, in particular, the critical role of ceremony and symbols, and the focus on developing professionalism and physician identities. There were also differences between them, in particular, in the way that the discourse in the literature focused on the symbolic and professional aspects of orientation; something we have called ‘cultural orientation’. Meanwhile, those who were responsible for orientation in their own institutions tended to focus on the practical and social dimensions.

**Conclusion:**

By examining how orientation has been described and discussed, we identify three domains of orientation: cultural, social, and practical. These domains are relatively distinct in terms of the activities associated with them, and in terms of who is involved in organizing and running these activities. We also describe orientation as a liminal activity system on the threshold of medical school where incoming students initially cross into the profession. Interestingly, this state of ambiguity also extends to the scholarship of orientation with only some of its aspects attracting formal enquiry, even though there is a growing interest in transitions in medical education as a whole. We hope, therefore, that this study can help to legitimize enquiry into orientation in all its forms and that it can begin to situate the role of orientation more firmly within the firmament of medical education practice and research.

## Introduction

Medical education research is a maturing discipline with an ever-growing evidence base for its many activities, processes, and systems. Transitions between pre-clerkship and clerkship have received a fair amount of attention (1, 2), as have transitions from MD training to residency (3). However the current literature reflects an apparent limited research interest in the design, practice, or experience of orientation activities for incoming medical students beyond the archetypal ‘white coat ceremony’ (4). This would seem to reflect a sense that orientation is neither fully within nor entirely outside of medical education and as such, enquiry into this time of transition faces questions of legitimacy in medical education scholarship. Nevertheless, for some institutions at least, orientation is valued as a significant part of the student experience and is an important part of the academic calendar. For these institutions, including the authors’ own schools, the question is not whether orientation is worthy of research but rather how should research be pursued in and around orientation practices and experiences.

This paper reports on a formative study that explored the potential research context of medical school orientation by comparing and contrasting existing discourses from the literature and from peer medical schools to develop a descriptive framework of key issues in medical school orientation thinking and practice. In doing so, we sought to take a critical stance on how orientation to medical school is understood, and the role of scholarship in shaping orientation and linking it to medical education as a whole.

## Background

Our approach to this study should be understood in the context of our affiliation with the Northern Ontario School of Medicine. Our charter class students created a commemorative yearbook for their graduation that contained many comments and photographs harking back to their orientation program 4 years earlier. Knowing the depth and breadth of educational experiences the students had had since orientation, we were quite surprised that their orientation experiences remained such a positive point of reference for these soon-to-be residents. In our efforts to better understand and enhance our own orientation program, we began to analyze the original assumptions that defined the orientation model we used, which led us to explore how orientation was perceived and practiced in other medical schools.

We approached medical school orientation as one of the ‘sentinel events and experiences that accelerate the attachment of professional identity’ (5), not least because professional identity formation has been shown to be significantly impacted by students’ earliest experiences (6).

We conducted a longitudinal mixed methods study to document, appraise, and explore the nature of orientation activities for students entering our own institution, a key part of this study was a review of what orientation meant to others both in the literature and at peer institutions. This paper reports on this latter contextual component of the study.

Our questions for the component of the study reported in this paper were:

1. What constitutes current practice in medical school orientation and how are they represented?
2. What does a research space for exploring orientation in a meaningful and productive way look like?

We operationalized these questions by comparing and contrasting existing discourses from the literature and from peer medical schools to develop a descriptive framework of key issues in medical school orientation thinking and practice. In doing so, we sought to take a critical stance on how orientation to medical school is understood, and the role of scholarship in shaping orientation and linking it to medical education as a whole.

## Methods

There were two data components to this review:

1. A literature search was conducted in 2011 using CINAHL and PubMed looking at the published literature from January 1, 2000, to December 31, 2010, using the terms ‘(medical OR health) AND student AND orientation’. The bibliographies of the 18 most relevant papers were used to identify other sources for the review. The material we identified contained a high proportion of opinion and argumentation and very little empirical data. We decided to use Gee's model of discourse analysis as the basis of a thematic synthesis both of what was discussed and how it was discussed (7, 8). This involved iteratively identifying and coding discursive themes in the texts around concepts of significance, practices, identities, relationships, politics, connections, and sign systems. The analysis was primarily conducted by one of the research teams (RHE) using standard coding techniques to develop a candidate model of the discourses in the literature around orientation to medical school. The other members of the team then reviewed this model, and consensus was iteratively built to create the final model we report in the results section of this paper.
2. A survey-based review of orientation activities in Canadian medical schools was undertaken. All 17 medical schools in Canada were approached to participate in this study. A pro forma survey was sent by email to the leads of student affairs department of each medical school in August 2011 – see Table 1. Reminder email messages were sent later in August and again in December 2011. Gee's model of discourse analysis was also applied to this data set.

**Table 1:** Survey questions; an accompanying rubric of issues and additional questions has been provided for each question

Question	Notes
Name of institution	Used to profile institution by size, culture, and so on.
Can you describe what essential factors or values make your program distinct?	Anticipating that these would be reflected in their orientation activities.
Can you describe the orientation process you provide for incoming students, in particular, how long does it last, what key events are there, how does it (if at all) articulate with the general university orientation?	Anticipating that a narrative account of what happens or at least what is legitimate and worthy of being reported would be shared.
Can you describe who is responsible for orientation in your school, who takes the lead role?	Anticipating that this may be organized by the university, the med school, the UG program, student societies, or some other entity or group.
What resources do you have available to run the orientation?	Anticipating that schools would be able to specify the human and fiscal resources allocated to orientation activities.
What are the key objectives for your orientation program?	Anticipating that only some institutions had specific objectives.
Who are the key stakeholders for your orientation program?	Anticipating that descriptions would reflect inclusiveness of patients, communities, other health professions, faculty, staff, family, friends, and existing students.
How does your orientation process reflect the needs of your students?	Anticipating that schools would describe a range of approaches that would relate to students’ needs, at least to some extent.
Can you describe how you evaluate and otherwise ensure the quality of your orientation process?	Anticipating that only some institutions include some component of evaluation or quality assurance.
Do you have any other comments or observations to share with us?	Anticipating that other issues are of importance to different schools.

The Research Ethics Boards of Lakehead University and Laurentian University approved this study.

## Results

*Literature search*: The CINAHL search identified 84 papers; however, few were specific to medical education (the majority being nursing related), and none were directly relevant to the study. The PubMed search identified 18 papers of which 15 were identified as relevant to the study. Another 26 papers were identified from the references given in the first trawl and included in the review. The literature we reviewed was dominated by reports and reflections on ‘white coat ceremonies’ (WCCs). Approximately, one-third of US medical schools (9) and just under two-thirds of Canadian medical schools have run some kind of WCC (1).

*Survey*: Eleven of Canada's 17 medical schools responded to our survey (64.7%), one of which was a francophone school (of 3 in Canada). Two schools elected to respond in the form of a semi-structured interview conducted by telephone while others completed a written report. It should be noted that although Canada has both English and French medical programs, for practical reasons the survey and literature searches were conducted only in English. Responding schools ranged in size and had no particular geographical concentration. Respondents were senior staff or leads from student affairs departments. Responses varied in length between 96 and 1,325 words with a mean of 526 words and a median of 247 words. The orientation activities they described ranged from 3 to 11 days in length, with a mean of 5.4 days and a median of 5 days. Although this was a convenience sample, we were satisfied that it was representative of Canadian schools in general for the purposes of discourse analysis.

### Discourses of orientation in the literature

We organized our interpretations using Gee's framework of discourse analysis:

1. *Significance:* the dominant discourse of orientation in the literature focused on the contestation of both what orientation should involve and what it should not, largely in the form of debate focused on the WCC as an ideal, and to an extent, abstract representation of the core values and philosophy of medical practice (4). Although closely connected with orientation, WCCs were also conducted at other times in medical programs, such as a part of the transition to the clerkship years (10). Some defended the white coat itself as a symbol, something ‘cloaked in magic’ (11), while others expressed concerns that the symbol of the white coat diminished the development of ‘virtuous physicians’ (12, 13). A particularly dominant theme in this discourse was incoming students’ adoption of a physician identity, most often with dimensions of professionalism, social responsibility, and ethics, the consideration of the privilege, authority, and responsibility that accompanies the physician identity were more often contested (14, 15). Developing a physician identity was discussed as intrinsically desirable, difficult to achieve (although still achievable through hard work and self-sacrifice), and as a mystery to which students are inducted. This discourse focused on orientation as an initiation and transformational process rather than knowledge transfer. Despite its presence in the literature, only 5 out of the 11 Canadian schools we surveyed made specific mention of some type of solemn ceremony, including a stethoscope ceremony, a Hippocratic oath ceremony (inclusive of a reading of the student code of conduct), an anatomy memorial service, and an oath ceremony. Only one school specifically used the term ‘white coat ceremony’.
2. *Ceremonies:* the setting for orientation activities was typically formal and solemn with most of the practices within them being ceremonial in nature; donning a white coat, reciting an oath, being addressed by dignitaries with inspiring and humbling words (16).
3. *Identities:* there were two primary identities discussed; the to-be-changed identity of the incoming student and the somewhat abstract identity of the idealized professional physician. The former being molded to take on the characteristics of the latter. While the physician identity was undeniably present, patients were typically not directly reflected in discourses in orientation activities although they were suggested in the discussion of professionalism and ethics.
4. *Relationships:* although students were the major focus, orientation was also identified as being beneficial to faculty in refreshing their commitment to teaching as it reinforces for them the special nature of their work (16). Students’ family and friends were also involved as witnesses for at least some of the proceedings. Carefully controlled socialization between incoming students and senior figures was another recurring feature (17).
5. *Politics:* orientation was a contentious topic, with a great deal of debate around the merits and weaknesses of the WCC (18–20). Some praised the WCC (15, 16, 21–23) as positively addressing the same issues that other criticized it for (20, 24, 25). The common themes in the discourse (identity, professionalism, ethics, and responsibility) were fairly constant; the interpretation of how different activities addressed them was the varying factor. This debate had grown to include other health professional education programs, in particular nursing (26–28). The articulation of the discourse was mostly narrative based and rhetorical with little substantive empirical evidence provided on either side of the debate.
6. *Connections:* although WCCs have typically been described with little specific connection to subsequent teaching and learning activities, there have been examples of how a WCC was connected to teaching around professional responsibilities (29) and professional identities (30, 31). Keirns et al. (10) also linked WCCs to professionalism and ethics teaching.
7. *Sign systems and knowledge:* archetypal symbols of the medical profession were common to much of this discourse, in particular the white coat and the ceremonies built around it. The oath was another key symbol in the discourse, usually presented as a defining threshold or transformational act for the student (14). Although medical professionalism and the ideal physician identity were discussed as constants in many of the papers reviewed, they also appeared to be somewhat contentious and were the main topics around which different ideal models of orientation were proposed and debated.

### Discourses of orientation practices in Canadian medical schools

These were also organized using Gee's framework of discourse analysis:

1. *Significance:* we identified three distinct organizing discourses of orientation activities across all responding institutions:
  - *Cultural orientation:* ceremonies and motivational presentations introducing students to the institutional and medical cultures of their program were common to all schools’ orientations, often with connotations of solemnity, responsibility, and initiation. Although cultural orientation activities seemed to be the minority in most schools’ approaches, their significance in the discourse was greater than that of the social or practical aspects of orientation.
  - *Social orientation:* is about binding individual students together as a coherent class unit. This was discussed as an intrinsically desirable outcome although it was unclear whether this was for the benefit of the student or the institution, or both. A key concept within social orientation was that of ‘fun’, often with students’ enjoyment of participating in group activities leading them to coalesce as a class. A discourse of socialization was also reflected in the number of activities that took place in private homes or other venues outside of the medical school. Social orientation appeared to be less significant than cultural orientation but more significant than practical orientation. Moreover, the significance of social orientation was greater for schools with distributed campuses due to perceived complications of identifying and bonding with peers from the same site. Schools with distributed programs (multiple teaching sites separated by significant distances) also tended to allude to the benefits of engaging the communities they served – essentially orienting to both school and community. For some, this created tensions that questioned the primary objective(s) of the orientation process.
  - *Practical orientation:* orientation is also used to provide practical information including how the curriculum is structured, how to succeed as a student, how to access student support resources, and introductions to the university and community context. Although the practical aspects of orientation took up a sizable proportion of the orientation schedule, they were perceived as being less significant than cultural or social orientation activities.
2. *Practices:* orientation activities aligned with the three axes identified in the previous section:
  - *Cultural orientation activities:* included WCCs and other ceremonies based around other symbols (e.g., the stethoscope), the community, and anatomy cadavers. A common component was the recitation of an oath. Motivational talks addressed topics such as professionalism and responsibility.
  - *Social orientation activities:* included ‘Med Olympics’ and team building survival games as well as ‘fun’ activities typically run by student organizations. These included visits to the park or beach, talent shows, and pub nights. Two schools mentioned that due to previous issues linked to alcohol misuse, students were reminded of their obligations associated with their student codes of conduct in the context of any activity that might involve alcohol in some way or the other.
  - *Practical orientation activities:* included tours of the campus, local community and/or the regional catchment area served by the school, as well as briefings and presentations from various service providers.
3. *Identities:* although the incoming class was mostly discussed as a homogeneous group, there was some recognition of actual or perceived differences between incoming students (such as maturity, prior experience, or social confidence) and the need to make sure they were not barriers to participation and engagement. Faculty and leadership (and administrators for practical aspects) and selected current students were the groups ‘delivering’ the orientation to the incoming students, and potentially benefitting from so doing.
4. *Relationships:* although not explicit in all circumstances, there seemed to be cycles of responsibility for orientation moving back and forth between students and the school, with conflicts between student centredness and institutional risk management being the main drivers for change.
5. *Authority and control:* responsibility for organizing orientation was a common theme in the discourse. For instance, five schools had student affairs offices (or their equivalents) taking the lead, while orientation at two other schools was organized by their undergraduate medical education offices. Five schools indicated that their orientation was run at the school level with little or no direct involvement of their undergraduate medical education offices. This indicated that, for these schools at least, orientation was more about joining a school and a profession than it was to joining a specific program of study. Cost was a sensitive issue with many respondents unwilling or unable to disclose specific budgetary details for their orientation activities, while others indicated that nobody had taken the time to estimate all the ‘under the table costs’ such as staffing and room utilization. Five schools did provide a global cost for orientation. These ranged from $1,000 to $100,000. Three schools asked their students to pay an additional fee (ranging between $50 and $150) to help offset the cost of orientation programming. This indicated a common discourse around institutional commitment and legitimacy. All of the responding medical schools ran their orientation activities more or less independent of their parent universities, thereby reinforcing the ‘other’ status of the medical school and its activities.
  Planning for orientation was another activity discussed by respondents with a common theme of inclusiveness; all but two schools involved students in the organization and execution of their orientation activities. That said, there was considerable variability regarding leadership/responsibility for orientation programs (student affairs, UME offices, students themselves). The issue of who leads the orientation program has important implications given the history of unfortunate events tied to these kinds of programs at post-secondary institutions (32).
6. *Connections:* the connections between orientation and the curriculum did not form a major part of the discourse, not least because of the focus on professional and institutional orientation rather than program orientation. Several schools described their curricula as being in a state of flux and although not explicit it was intimated that this created an ongoing disconnect between orientation and the curriculum. Although discussion of budgets was limited, sponsorship and the connection to particular extra-program entities was a recurring theme. Sponsorships were mostly from departments within the medical school (e.g., family medicine) or from the university as a whole, although they could also come from external sponsors in the community. It was notable that all of the schools surveyed ran separate orientation programs from their host universities thereby minimizing student exposure to non-medical school perspectives and maximizing their immersion to the culture of medicine.
7. *Sign systems:* different schools used different names for their orientation programs, although their components were often quite similar. Locally significant names for events were common, often linking to the history or mission of the institution. Symbols of the medical profession were common to all schools although in different forms, such as white coats and stethoscopes. Nurturing symbols were also common, in particular food, either formally as in catered presentations or in more informal/social settings like barbecues. The creation of artifacts and archives capturing the orientation experience was important for some schools but not for others.

### Synthesis of discourses of orientation practices

The findings from the two discourse analyses were compared to identify converging and diverging themes – see Table 2. Of particular note were the ceremonial and archetypical aspects of orientation that were to be found in both discourses, and the practical and program-related aspects of orientation which were only identified in the discourse from the medical school survey respondents.

**Table 2:** Comparison of convergent and divergent themes from the discourses of orientation in the literature and from the Canadian medical schools that responded to the survey

Discourse dimension	Converging themes	Diverging themes
Significance	Incoming students’ adoption of a physician identity, including professionalism, social responsibility, and ethics. Orientation as initiation and transformation more than knowledge transfer.	Contestation of both what orientation should involve and what it should not. The role of social orientation, class bonding, and students as organizers.
Practices	Ceremonies; donning a white coat, reciting an oath, being addressed by dignitaries.	Social orientation activities, team building, and ‘fun’ activities. Practical orientation activities, including tours and briefings. Planning involving students and service providers.
Identities	The to-be-changed identity of the incoming student and the abstract identity of the idealized professional physician. Faculty and leadership as role models. Friends and family as witnesses.	Involvement of communities in planning and running orientation activities. Community involvement might lead to questions regarding the primary objective(s) of the orientation process.
Relationships	[No common themes]	Benefits to faculty. Responsibility for orientation moving back and forth between students and the school.
Politics	[No common themes]	Debate around the merits and weaknesses of the WCC. Orientation more about joining a school and a profession than joining a specific program of study. Cost of organizing and running orientation.
Connections	Focus on professional and institutional orientation rather than program orientation.	Sponsorship from internal and external units and organizations. Curricular change creating disconnect between orientation and the program the students are entering.
Sign systems	Archetypal symbols of the medical profession, white coats, stethoscopes. An oath as a threshold or transformational act.	Orientation as a battleground of the reproduction or alteration of physician identity. Use of names linked to the history or mission of the institution. Nurturing symbols, in particular food. Artifacts capturing the orientation experience.

## Discussion

We have structured our discussion of our findings around our two research questions:

### Current practice in medical school orientation

Orientation to medical school would seem to vary between institutions although it does seem to involve many common component activities and philosophies. This was reflected in the similarities between the two discourses of orientation that we analyzed, in particular the critical role of ceremony and symbols and the focus on professionalism and physician identity development. There were also differences in the way that the discourse in the literature remained focused on the symbolic and professional aspects of orientation, something we have called ‘cultural orientation’. The survey identified two other forms of orientation practice: ‘social orientation’ intended to bind individual students together as a coherent and supportive class, and ‘practical orientation’ briefing students on the basic facts needed to function in the program and to process various administrative tasks.

Orientation was typically defined as a series of highly situated events that took place shortly before, at, or shortly after a class of medical students started their studies. These events involved aspects of social, practical, and cultural orientation to what it is to be a medical student in a particular place at a particular time. The focus on the class, rather than the individual, would seem to be a deliberate, if a less than clearly articulated, mechanism that takes a group of individuals and socializes them so as to unify them as a class.

A key difference between the two discourses was that, while student involvement in designing and leading orientation activities was identified by a number of survey respondents, it was largely absent from the literature. The primary reason for this would seem to be linked to senior students’ focus on the social rather than the professional dimensions of orientation. Furthermore, although some of the survey respondents discussed the involvement of their local communities in their orientation activities, we were unable to find equivalent references to such practices within the literature, despite a growing focus on social accountability in medical education as a whole (33).

The variety of institutional views on the legitimacy and significance of orientation was reflected in the levels and forms of funding for orientation, and in the shifting balance between student and institutional responsibility for orientation. It would seem that, in an age where professionals are expected to be ever more abstemious and accountable for their actions, the acceptability of alcohol and hazing in orientation has diminished and risk management has become an increasing part of orientation planning for medical schools. As a result, orientation has become more of an institutional responsibility over time and would seem to be likely to continue in this direction for some time to come.

### Developing a research space for exploring medical school orientation

**Fig. 1:**
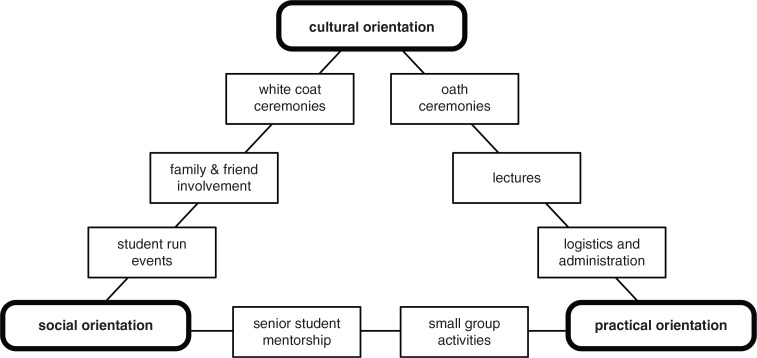
Essential activities of orientation organized according to their involvement of social, cultural, or practical dimensions of orientation.

Our work has identified several dimensions of potential research in and around medical school orientation:

1. Medical school orientation may be the subject of research in and of itself as a social and logistical phenomenon. The three dimensions of cultural orientation, social orientation, and practical orientation we identified in the current study can form a single theoretical framework of orientation practices – see Fig. 1. This could serve as a program theory for future enquiry into this as-yet under-researched aspect of medical education. Other areas of potential inquiry include economics, stakeholder representation, discourses within the conduct of orientation, and participant outcomes.
2. Orientation may also be researched as the first of many stages in medical students’ training. For instance, a class is the organizational unit with which researchers typically work, yet its origins are rarely if ever considered. Understanding how a class forms in orientation could be expected to provide important insights on how the individual and collective identities of the class may subsequently respond to different educational interventions.
3. Orientation may be researched as a construct within a broader educational ecology. For instance, although orientation has no explicit assessment, there is an implicit assessment as individual student's ability to benefit from orientation may well impact their ability to succeed later in their studies. Research may also consider more theoretical constructs, such as the hidden curricula of orientation, the impact of orientation on learner identities, or orientation to their medical school's communities of academic and clinical practice. The need for further research in this latter area was implied by the ways that our findings echo Wenger's models of ‘communities of practice’ (34), such as the roles of novitiates and those that induct them into a community of practice, the role of shared symbols and boundaries for a community of practice, and the ways in which these boundaries can be legitimately crossed.
4. Orientation sits at the threshold of medicine and could be researched as a liminal time when students are neither in nor out of the medical school or medical profession, and where they start to set aside aspects of their previous lives as they begin their journey to become physicians. This observation parallels Turner's work on rituals (35, 36), which he describes as liminal socially complex activities required for individuals to cross social thresholds. Although the discourse on orientation in medical education often uses the term ‘ceremony’, a small but significant part of orientation activity involves ritual, at least in following Turner's observation that while ‘ceremony indicates, ritual transforms’ (35, p. 80).
5. Orientation may also provide a valuable professional development opportunity for existing students by providing opportunities for them to plan, manage, and lead orientation activities, and to mentor incoming students. The potential for participation in orientation to develop leadership, organizational, and mentorship skills raises the possibility of researching it as an educational intervention for these students. independently or in tandem with its impact on incoming students.

Orientation is, as yet, an under-investigated aspect of medical education practice, and there are many ways in which research in and around orientation could be developed and conducted. Our own lines of inquiry include aspects of the hidden curriculum and identity formation in our own institution's orientation. We hope to learn from others’ explorations in to different aspects of orientation at their own institutions.

### Limitations

We acknowledge a number of limitations to this study. First, we have addressed a very large (and somewhat under-explored) area of medical school practice and in doing so, we have chosen to review how orientation is discussed rather than directly observing orientation activities, sampling participant experiences, or describing or measuring the outcomes of such activities.

This was not an in-depth study as the literature search and survey were both limited in scope and rigor in the way they were planned and executed as we focused on developing a broad understanding of the concepts of orientation as a precursor to, and as a way of informing other parts of, our study into orientation at our own institution. This study is formative and question-generating rather more than it is question-answering a clear precursor, at least provisionally, for defining a research space.

On a more practical note, we acknowledge the limitation of only conducting the survey and literature search in English and only at Canadian schools. A more thorough review might have considered a wider range of different cultures (both social and geographical). However, as previously stated, this study was about defining a research space rather than fully occupying it, and as such the limited range of contexts we examined aligned with our own institution's setting.

The two discourses we decided to analyze were somewhat discontinuous. One pre-existed the study and was dominated by debate around a particular ceremonial set of practices and associated ideologies. The other was generated by the project with its format and focus predetermined and to some extent directing of the responses we entered into our analysis. Treating both as discourses allowed them to be synthesized but in doing so we acknowledge that there are limitations to the comprehensiveness of the findings we present.

We also acknowledge that our use of discourse analysis was relatively cursory compared to its use to analyze single texts in depth. This was a deliberate methodological choice as we used discourse analysis as a way of analyzing and synthesizing multiple academic papers and survey results in a single model. Discourse has proved to be a valuable technique to use, particularly where there is so much contention (in the literature at least) and the concept of orientation is so limited, neither fully within or outside legitimate scholarly enquiry. Our work is in itself, an act of exploring the legitimacy of inquiry in and around this topic and as such we acknowledge that it too will contribute to and be held accountable within the discourse of orientation to medical school.

## Conclusions

Orientation to medical school is a complex, culturally rich, and to some extent ritualistic undertaking, only part of which has been explored in the literature. This study has attempted to describe orientation to medical school by looking at how it has been described and discussed rather than looking at its practices or outcomes. In doing so, we have found that orientation has three domains, namely cultural, social, and practical, and that these domains are relatively distinct in terms of the activities associated with them, and in terms of who is involved in organizing and running these activities.

We have also been able to identify and describe orientation as a process of professional transition, both as an activity system that sits on the threshold of medical school and as a gateway to the medical profession for individual incoming students. This juxtaposition also extends to scholarship with only some aspects of orientation attracting formal enquiry even though there is a growing interest in transitions in medical education as a whole. We hope, therefore, that this study can help to legitimize enquiry into orientation in all its forms and that it can begin to situate the role of orientation more firmly within the firmament of medical education practice and research.

## References

[1] Hendelman W, Byszewski A (2007). A national survey: medical professionalism in Canadian undergraduate programs. http://www.afmc.ca/pdf/ppt_2007_professionalism_canada_en.ppt.

[2] Reznick R (2011). Ready to fly – dean on campus blog. http://meds.queensu.ca/blog/?p=1193.

[3] Schulz GM (2007). Improving your new resident orientation program. A perspective from the University of Missouri-Columbia. J Surg Educ.

[4] Arnold P, Gold Foundation (2012). White coat ceremony. http://www.humanism-in-medicine.org/index.php/programs_grants/gold_foundation_programs/white_coat_ceremony.

[5] Sklar DP (2013). Beginning the journey. Acad Med.

[6] Niemi PM, Vainiomaki PT, Murto-Kangas M (2003). “My future as a physician” – professional representations and their background among first-day medical students. Teach Learn Med.

[7] Gee JP, Green JL (1998). Discourse analysis, learning and social practice: a methodological study. Rev Res Educ.

[8] Gee JP (1999). An introduction to discourse analysis: theory and method.

[9] Russell PC (2002). The white coat ceremony: turning trust into entitlement. Teach Learn Med.

[10] Keirns CC, Fetters MD, De Vries RG, Brosnan C, Turner BS (2009). Bioethics and medical education: lessons from the United States. Handbook of the sociology of medical education.

[11] Druss RG (1998). The magic white coat. Ann Intern Med.

[12] de Marco DG (1999). Letter: contemplating the white coat. Ann Intern Med.

[13] Seigle SP (1999). Letter: contemplating the white coat. Ann Intern Med.

[14] Gillon R (2002). In defence of medical commitment ceremonies. J Med Ethics.

[15] Glick SM (2003). White coat ceremonies – another commentary. J Med Ethics.

[16] Huber SJ (2003). The white coat ceremony: a contemporary medical ritual. J Med Ethics.

[17] Spindler S (1992). Doctors to be.

[18] Blumhagen DW (1979). The doctor's white coat: the image of the physician in modern America. Ann Intern Med.

[19] Anvik T (1990). Doctors in a white coat – what do patients think and what do doctors do?. Scand J Prim Health Care.

[20] Russell PC (2002). The white coat ceremony: turning trust into entitlement. Teach Learn Med.

[21] Paulsen MM (1999). Letter: contemplating the white coat. Ann Intern Med.

[22] Branch WT (1998). Deconstructing the white coat. Ann Intern Med.

[23] Branch WT (1999). Letter: contemplating the white coat. Ann Intern Med.

[24] Wear D (1998). On white coats and professional development: the formal and the hidden curricula. Ann Intern Med.

[25] Veatch RM (2002). White coat ceremonies: a second opinion. J Med Ethics.

[26] Connelly LM, Hoffart N (1998). A research-based model of nursing orientation. J Nurs Staff Dev.

[27] Ragsdale M, Mueller J (2005). Plan, do, study, act model to improve an orientation program. J Nurs Care Qual.

[28] Altman MI, Musselman M, Curry L (2010). Success begins in nursing freshman orientation course. Nurs Educ.

[29] Rhodes R (2001). Enriching the white coat ceremony with a module on professional responsibilities. Acad Med.

[30] Cohn F, Lie D (2002). Mediating the gap between the white coat ceremony and the ethics and professionalism curriculum. Acad Med.

[31] Elcin M, Odabasi O, Gokler B, Sayek I, Akova M, Kiper N (2006). Developing and evaluating professionalism. Med Teach.

[32] Allan EJ Hazed and confused: transforming hazing cultures. http://www.npr.org/programs/atc/features/2005/nov/hazing/allannewsletter.pdf.

[33] Woollard R, Boelen C (2012). Seeking impact of medical schools on health: meeting the challenges of social accountability. Med Educ.

[34] Wenger E (1998). Communities of practice.

[35] Turner V, Moore SF, Myerhoff BG (1977). Variations on a theme of liminality. Secular ritual.

[36] Turner V (1982). From ritual to theatre.

